# Perspectives on Sex- and Gender-Specific Prediction of New-Onset Atrial Fibrillation by Leveraging Big Data

**DOI:** 10.3389/fcvm.2022.886469

**Published:** 2022-07-11

**Authors:** Sven Geurts, Zuolin Lu, Maryam Kavousi

**Affiliations:** Department of Epidemiology, Erasmus MC, University Medical Center Rotterdam, Rotterdam, Netherlands

**Keywords:** atrial fibrillation, artificial intelligence, big data, prediction, sex- and gender differences

## Abstract

Atrial fibrillation (AF), the most common sustained cardiac arrhythmia, has a large impact on quality of life and is associated with increased risk of hospitalization, morbidity, and mortality. Over the past two decades advances regarding the clinical epidemiology and management of AF have been established. Moreover, sex differences in the prevalence, incidence, prediction, pathophysiology, and prognosis of AF have been identified. Nevertheless, AF remains to be a complex and heterogeneous disorder and a comprehensive sex- and gender-specific approach to predict new-onset AF is lacking. The exponential growth in various sources of big data such as electrocardiograms, electronic health records, and wearable devices, carries the potential to improve AF risk prediction. Leveraging these big data sources by artificial intelligence (AI)-enabled approaches, in particular in a sex- and gender-specific manner, could lead to substantial advancements in AF prediction and ultimately prevention. We highlight the current status, premise, and potential of big data to improve sex- and gender-specific prediction of new-onset AF.

## Introduction

Atrial fibrillation (AF), the most common cardiac arrhythmia, markedly increases the risk of hospitalization, morbidity, and mortality (1–3). Over the last two decades advances regarding the clinical epidemiology and management of AF have been established (1–3). Recent evidence indicates that both sex and gender play a role in the development and progression of cardiovascular disease, drug reactions, and healthcare utilization (4–7). While sex refers to the biology; including chromosomes, gene expression, hormone levels, and their function, gender comprises the socio-cultural attributes; including socially constructed roles, behaviors, expressions, and identities (4–7). In the field of AF, however, sex and gender implications remain understudied (2, 8–10). Notably, the age-adjusted prevalence, incidence, and lifetime risk of AF are higher in men than in women (1–3). More specifically, it is suggested that several risk factors (hypertension, smoking, alcohol intake, obesity, history of diabetes mellitus, history of myocardial infarction or history of heart failure), carry a differential impact on AF risk in men and women (1, 8–11). It has also been suggested that AF-related adverse outcomes and response to various treatment modalities differ between men and women (1–3, 8–11). Nevertheless, AF remains to be a complex and heterogeneous disorder and a comprehensive sex- and gender-specific approach to predict new-onset AF is lacking.

The past decade has witnessed an exponential growth in recorded data in the healthcare sector. The massive amount of recorded information, i.e. big data, has turned to a topic of special interest, because of its great potential. Leverage of big data, using artificial intelligence (AI)-enabled approaches, provides an opportunity to further improve prediction of AF (12, 13). Use of various sources of big data such as electrocardiograms (ECGs), electronic health records (EHRs), and wearable devices, in particular in a sex- and gender-specific approach, could lead to substantial advancements in AF prediction and ultimately prevention. Here, we review the current status and highlight the premise and potential of these data sources to improve sex- and gender-specific prediction of new-onset AF.

## Leveraging Big Data for Prediction of New-onset Atrial Fibrillation

Over the past decade, several AF risk prediction scores have been developed and validated using more traditional research methods (14–22). These prediction scores predominantly use traditional cardiovascular risk factors such as age, sex, race, height, weight, hypertension, diabetes mellitus, coronary heart disease, and heart failure which are readily obtainable clinical variables (14–22). In general, these scores have a moderate to good performance (C-statistic ranging between 0.65–0.78) (14–22). Over the past years, there has been an exponential interest in using various big data sources to further improve the AF risk prediction beyond the traditional AF risk factors (23, 24). Specifically, multiple studies have employed AI-enabled algorithms to evaluate new-onset AF prediction by leveraging various big data modalities including the clinical data, ECGs, EHRs, and wearable devices (23–42). Some of these studies showed that AI-enabled AF prediction models performed similar to or better than established traditional AF prediction models (25, 27–30). Furthermore, targeted AF screening using a machine learning (ML) risk prediction algorithm showed the potential to enhance AF screening and to improve the cost-effectiveness of AF screening through an efficient use of limited healthcare resources (31).

A variety of studies have highlighted the potential predictive capacity of AI to assess the risk of new-onset AF from a 12-lead ECG with acceptable to excellent performance (area under the receiver operating characteristic curve ranging between 0.70–0.90) (32, 33). The potential of the ECG to predict AF might be explained by the fact that the AF substrate is caused by electrical and structural remodeling of the heart (2). AI-enabled algorithms for ECG assessment could mark the very early stages of remodeling, not yet being detected by the cardiologist using routine measures, to predict new-onset AF. However, previous studies mainly predicted new-onset AF risk within a short time period (<1 year) (33). Thereby, their value for primary prevention is limited, as the time window may not be long enough to intervene in individuals at high AF risk. In a recent study (32), a convolutional neural network for 10-second ECG measures was trained to infer 5-year risk of new-onset AF. The investigators concluded that their method had similar predictive utility as the widely accepted clinical risk factor model: the Cohorts for Heart and Aging Research in Genomic Epidemiology AF score (CHARGE-AF) (14). Yet, the combination of both (clinical risk factors and ECG) provided the greatest predictive accuracy with good discrimination and calibration. These findings underscore the capacity of AI-enabled approaches to improve AF prediction in an inexpensive, non-invasive, widely available, and point-of-care testing manner.

Two studies utilized EHRs to develop AF prediction models (41, 42). EHRs contain real-time, patient-centered data that are instantly available to patients and authorized healthcare providers. The increasing digitalization of healthcare systems makes EHRs more and more widely available, making them particularly useful to predict new-onset AF. A recent study developed an AF prediction model for a 6-month time period using 200 most common EHR features of 2,252,219 individuals. However, this ML approach did not substantially perform better than a logistic regression model using traditional AF risk factors (41). Another study developed a model to predict new-onset AF over a 2-year time period with good discrimination (C-statistic of 0.81) using a 10-variable model compromised of covariates commonly available in the EHRs of 53,552 subjects. This study, however, did not compare the developed prediction model to previous validated AF risk prediction models (42). Another study evaluated the improvement in 5-year AF risk prediction when adding novel variables identified by ML to the CHARGE-AF enriched score (27). Although this study was not conducted using EHRs, it did include clinical, serological, echocardiographic, and cardiac imaging information that are increasingly becoming more available within EHRs. This method, however, did not significantly improve AF prediction in comparison to the CHARGE-AF enriched score (27).

The emergence of wearable devices constitutes another major source to improve AF prediction and management (23). Wearable devices often use photoplethysmography or ballistocardiography to monitor an individual's cardiac rhythm. Other forms of wearable devices include cardiac implantable electronic or patch-based ECG devices that have proven to be useful in selected patient populations to detect AF or assess the risk of stroke in AF patients beyond the CHA_2_DS_2_-VASc score (43, 44). Such wearable devices are simple to apply and enable real-time continuous monitoring of the heart. This makes the use of wearable devices promising as one could use this electrical information to predict new-onset AF. Nonetheless, the use of wearable devices and AI algorithms is currently mainly limited to AF detection rather than new-onset AF prediction.

## Premise of Big Data in Prediction of New-Onset Atrial Fibrillation

Leveraging big data by AI-enabled approaches could offer great opportunities to improve AF prediction. First, AI methods could be used to overcome statistical issues that are potentially challenging in traditional approaches. In particular, for complex diseases such as AF, simultaneous use of hundreds of quantitative biomarkers may lead to problems such as multi-collinearity, non-linearity, complex interactions, and the possibility of over-fitting (45–47). AI methods, including random (survival) forest method, a non-parametric ML decision tree-based approach, have been proposed to overcome such challenges (45, 47). Second, AI approaches allow for data mining purposes to automatically extract more valuable information from unstructured and complex datasets to improve AF prediction. Ultimately, use of AI would allow combination of various extensive, annotated data libraries into multidimensional datasets which include genotyping, imaging, clinical, and other subphenotypic information. These multidimensional datasets could be used to identify different AF subphenotypes which then could be utilized to improve AF prediction, prevention, and management in a potentially sex and gender-specific manner. Particularly, the inclusion of multilayered high-throughput omics data in such datasets seems promising, given the vast contribution of genetic studies (genome-wide association studies, experimental and in silico candidate gene studies, and Mendelian randomization studies) to advance AF pathophysiology. Recently, a data-driven cluster analysis of 9,749 AF patients, using 60 clinical characteristics, led to identification of 4 cluster AF phenotypes (48). The 4 AF phenotypes were: AF with limited risk factors, younger AF patients with comorbid behavioral disorders, AF patients with tachycardia-bradycardia with device implantation due to sinus node dysfunction, and AF with atherosclerotic vascular disease (48). Another cluster analysis of 2,458 AF patients, using 46 variables, identified 3 cluster AF phenotypes including younger paroxysmal AF, persistent/permanent AF with left atrium enlargement, and atherosclerotic comorbid AF in elderly (49). Another study used hierarchical clustering analyses to identify distinct phenotypes of primary mitral regurgitation which is also considered a heterogeneous disease, as it is the case with AF (50). As such, AI applications could further aid in improving subphenotypic AF classifications to further unravel the complexity and heterogeneity of AF. Based on data-driven approaches, rather than hypothesis-driven approaches with a-priori assumptions, leverage of big data with AI methods can also identify and prioritize AF biomarkers within the realm of AF risk prediction.

## Potential of Big Data for Sex- and Gender-Specific Prediction of New-onset Atrial Fibrillation

While big data sources such as ECGs, EHRs, and wearable devices could improve AF prediction and management, their potential for a sex- and gender-specific approach to AF needs further attention.

In particular, recent electrophysiological evidence highlights sex differences with regard to cardiac cellular electrophysiology and their translation to the ECG parameters. It is well documented that sex hormones affect the action potential morphology and cellular electrophysiology through their influence on ion channel function and current densities. Specifically, men have a shorter action potential duration, have a more prominent phase 1 repolarization, and shorter phase 3 repolarization than women (51). It is hypothesized that primarily inward depolarizing L-type Ca^2+^ current and outward repolarizing K^+^ currents modified by sex hormones are responsible for these sex differences in action potential morphology. Moreover, women have a higher heart rate, lower heart rate variability, shorter and taller P waves, shorter PR interval, shorter and smaller QRS complexes, longer QT and QTc interval, longer JT interval, wider and smaller T waves, smaller J point, and smaller ST segment in comparison to men (51). From a clinical perspective, the longer QTc interval in women makes women more prone to drug-induced QTc prolongation which may result in torsades de pointes (51). The higher J point and ST segment in men may explain the higher prevalence of J-wave syndromes (Brugada syndrome, and early repolarization syndrome) in men (51). Next to these sex hormones' modulations on a cellular level, it is thought that the smaller size of the women's heart, at least partially, explains some of the documented ECG sex-differences. Noteworthy, these sex differences in sinus rhythm generally persist when men and women develop AF (52).

EHRs hold real-time, patient-centered data from men and women that reflect sexual and biological differences such as obesity and hypertension, among others. However, lack of precise and inclusive documentation of gender, the socio-culturally constructed characteristics of men and women, in EHRs is notable (7, 53). Although, gender documentation may be incomplete in the narrow sense (no actual documentation of i.e. cis-, trans-, or non-binary gender identity), EHRs provide more information in a broad sense on socio-cultural habits, thereby representing gender. More specifically, EHRs could provide information on socio-culturally determined characteristics, roles or habits; including but not limited to socio-economic status, physical and social behaviors that influence e.g. physical activity, social interaction, medication use and adherence to medication, and healthcare use (5–7). Such information could shed more light on gender-related factors that may impact AF. With regards to wearable devices, previous evidence has shown a higher burden of atrial and ventricular arrhythmia in women using a wearable cardioverter-defibrillator, compared to men (54). This is in line with the evidence underpinning the existence of sex-specific ECG features, as mentioned earlier. In addition, similar to EHRs, wearable devices may give more insight into gender-related behavioral habits including physical activity, working hours, sleep, caloric and fluid intake, and other social activities. The latter variables have indeed been suggested to be of added value when modeling sex and gender differences in various health domains, although data to support this claim within the AF field is lacking (55).

The sex-related dimensions in ECGs, and gender-related features in EHRs and wearable devices, as discussed earlier, are recognizable and should yet be fully explored to improve AF prediction and management. AI-enabled algorithms may be able to detect more sex-specific characteristics and gender-related behavioral patterns within these big data sources that might not be apparent on a more macroscopic level or by using traditional statistical methods. This may eventually improve our understanding of sex- and gender-related differences in AF.

## Discussion

This review highlighted the current status, premise, and potential of big data to improve prediction of new-onset AF in a sex- and gender-specific manner. While leveraging big data using AI-enabled algorithms offers major opportunities to further advance AF prediction, adoption of a sex- and gender-specific approach is still lacking.

Ample challenges remain before AI-enabled algorithms can be adopted for prediction, prevention, and management of AF. First, the interpretability (transparency and explainability) of AI and exact definition of how all the different methods work is yet difficult (56). This so called “black box” is dependent on the type of AI algorithms that is being used (in particularly deep learning). Application of such algorithms warrants careful and thorough examination of the methods before their implementation, because an algorithm that is intransparent and/or unexplainable may lead to erroneous conclusions that could potentially harm a patient. Second, validation and calibration of AI-enabled algorithms for AF prediction while using external data sources are essential before such algorithms could be widely adopted, implemented, and used within the AF field. Third, the classification codes of clinical variables, drugs, and diseases in different countries and hospitals are different. This leads to challenges with regard to data extraction and harmonization. Careful standardization to harmonize the data, derived from multiple sources, and to integrate all the data modalities within a multidimensional dataset is warranted. Fourth, various ethical issues such as privacy, transparency, informed consent, and trust should be taken care of, and the potential for criminal and malicious use and contested ownership of data should be carefully considered (57). Lastly, rigorosity of AI-enabled algorithms depends on the objectivity, quality, and size of the data used to train them. False, low quality, non-representative study sample, and/or missing data will result in invalid models, while also limiting the generalizability of such models (58). This latter limitation is in particular of concern, as it further magnifies the sex and gender inequalities that already exist within research.

Large, diverse, and multidimensional data sources carry the potential to improve AF prediction and management. Yet, various challenges remain before AI-enabled algorithms can be adopted and implemented. Enhancement of personalized and precision medicine in AF warrants taking into account the complexity of sex and gender dimensions in big data sources and methods, while also overcoming the challenges that currently accompany the use of AI-enabled algorithms.

## Author Contributions

Conceptualization, project administration, and validation: SG and MK. Data curation, investigation, methodology, resources, writing—original draft, and writing—review and editing: SG, ZL, and MK. Funding acquisition and supervision: MK. Software and visualization: SG. All authors contributed to the article and approved the submitted version.

## Funding

This study was further supported by the Senior Scientist Grant from Dutch Heart Foundation (03-004-2021-T050).

## Conflict of Interest

The authors declare that the research was conducted in the absence of any commercial or financial relationships that could be construed as a potential conflict of interest.

## Publisher's Note

All claims expressed in this article are solely those of the authors and do not necessarily represent those of their affiliated organizations, or those of the publisher, the editors and the reviewers. Any product that may be evaluated in this article, or claim that may be made by its manufacturer, is not guaranteed or endorsed by the publisher.

## References

[1] Chugh SS Havmoeller R Narayanan K Singh D Rienstra M Benjamin EJ. Worldwide epidemiology of atrial fibrillation: a Global Burden of Disease 2010 Study. Circulation. (2014) 129:837–47. 10.1161/CIRCULATIONAHA.113.005119 24345399 PMC4151302

[2] Hindricks G Potpara T Dagres N Arbelo E Bax JJ Blomstrom-Lundqvist C. 2020 ESC Guidelines for the diagnosis and management of atrial fibrillation developed in collaboration with the European Association for Cardio-Thoracic Surgery (EACTS): the task force for the diagnosis and management of atrial fibrillation of the European Society of Cardiology (ESC) developed with the special contribution of the European Heart Rhythm Association (EHRA) of the ESC. Eur Heart J. (2021) 42:373–498. 10.1093/eurheartj/ehab648 32860505

[3] Krijthe BP Kunst A Benjamin EJ Lip GY Franco OH Hofman A. Projections on the number of individuals with atrial fibrillation in the European Union, from 2000 to 2060. Eur Heart J. (2013) 34:2746–51. 10.1093/eurheartj/eht280 23900699 PMC3858024

[4] Schirmer SH Hohl M Bohm M. Gender differences in heart failure: paving the way toward personalized medicine? Eur Heart J. (2010) 31:1165–7. 10.1093/eurheartj/ehq073 20304837

[5] Stolarz AJ Rusch NJ. Gender differences in cardiovascular drugs. Cardiovasc Drugs Ther. (2015) 29:403–10. 10.1007/s10557-015-6611-8 26227895

[6] Doull M Runnels VE Tudiver S Boscoe M. Appraising the evidence: applying sex- and gender-based analysis (SGBA) to Cochrane systematic reviews on cardiovascular diseases. J Womens Health. (2010) 19:997–1003. 10.1089/jwh.2009.1626 20384450

[7] Johnson JL Greaves L Repta R. Better science with sex and gender: Facilitating the use of a sex and gender-based analysis in health research. Int J Equity Health. (2009) 8:14. 10.1186/1475-9276-8-14 19419579 PMC2689237

[8] Kavousi M. Differences in epidemiology and risk factors for atrial fibrillation between women and men. Front Cardiovasc Med. (2020) 7:3. 10.3389/fcvm.2020.00003 32118043 PMC7025483

[9] Ko D Rahman F Martins MA Hylek EM Ellinor PT Schnabel RB. Atrial fibrillation in women: treatment. Nat Rev Cardiol. (2017) 14:113–24. 10.1038/nrcardio.2016.171 27786235 PMC5520636

[10] Ko D Rahman F Schnabel RB Yin X Benjamin EJ Christophersen IE. Atrial fibrillation in women: epidemiology, pathophysiology, presentation, and prognosis. Nat Rev Cardiol. (2016) 13:321–32. 10.1038/nrcardio.2016.45 27053455 PMC5579870

[11] Staerk L Wang B Preis SR Larson MG Lubitz SA Ellinor PT. Lifetime risk of atrial fibrillation according to optimal, borderline, or elevated levels of risk factors: cohort study based on longitudinal data from the Framingham Heart Study. BMJ. (2018) 361:k1453. 10.1136/bmj.k1453 29699974 PMC5917175

[12] Niculescu V. On the Impact of High Performance Computing in Big Data Analytics for Medicine. Appl Med Inform. (2020). 42:9–18. Available from: https://ami.info.umfcluj.ro/index.php/AMI/article/view/766

[13] Lin S-H Ikram MA. On the relationship of machine learning with causal inference. Eur J Epidemiol. (2020) 35:183–5. 10.1007/s10654-019-00564-9 31560086

[14] Alonso A Krijthe BP Aspelund T Stepas KA Pencina MJ Moser CB. Simple risk model predicts incidence of atrial fibrillation in a racially and geographically diverse population: the CHARGE-AF consortium. J Am Heart Assoc. (2013) 2:e000102. 10.1161/JAHA.112.000102 23537808 PMC3647274

[15] Chamberlain AM Agarwal SK Folsom AR Soliman EZ Chambless LE Crow R. A clinical risk score for atrial fibrillation in a biracial prospective cohort (from the Atherosclerosis Risk in Communities [ARIC] study). Am J Cardiol. (2011) 107:85–91. 10.1016/j.amjcard.2010.08.049 21146692 PMC3031130

[16] Hulme OL Khurshid S Weng LC Anderson CD Wang EY Ashburner JM. Development and validation of a prediction model for atrial fibrillation using electronic health records. JACC Clin Electrophysiol. (2019) 5:1331–41. 10.1016/j.jacep.2019.07.016 31753441 PMC6884135

[17] Li YG Pastori D Farcomeni A Yang PS Jang E Joung B. A simple clinical risk score (C2HEST) for predicting incident atrial fibrillation in Asian subjects: derivation in 471,446 Chinese subjects, with internal validation and external application in 451,199 Korean subjects. Chest. (2019) 155:510–8. 10.1016/j.chest.2018.09.011 30292759 PMC6437029

[18] Schnabel RB Sullivan LM Levy D Pencina MJ Massaro JM D'Agostino RB. Sr.. Development of a risk score for atrial fibrillation (Framingham Heart Study): a community-based cohort study. Lancet. (2009) 373:739–45. 10.1016/S0140-6736(09)60443-8 19249635 PMC2764235

[19] Suenari K Chao TF Liu CJ Kihara Y Chen TJ Chen SA. Usefulness of HATCH score in the prediction of new-onset atrial fibrillation for Asians. Medicine. (2017) 96:e5597. 10.1097/MD.0000000000005597 28072697 PMC5228657

[20] Brunner KJ Bunch TJ Mullin CM May HT Bair TL Elliot DW. Clinical predictors of risk for atrial fibrillation: implications for diagnosis and monitoring. Mayo Clin Proc. (2014) 89:1498–505. 10.1016/j.mayocp.2014.08.016 25444486

[21] Cabrera S Valles E Benito B Alcalde O Jimenez J Fan R. Simple predictors for new onset atrial fibrillation. Int J Cardiol. (2016) 221:515–20. 10.1016/j.ijcard.2016.07.077 27414732

[22] Saliba W Gronich N Barnett-Griness O Rennert G. Usefulness of CHADS2 and CHA2DS2-VASc scores in the prediction of new-onset atrial fibrillation: a population-based study. Am J Med. (2016) 129:843–9. 10.1016/j.amjmed.2016.02.029 27012854

[23] Olier I Ortega-Martorell S Pieroni M Lip GYH. How machine learning is impacting research in atrial fibrillation: implications for risk prediction and future management. Cardiovasc Res. (2021) 117:1700–17. 10.1093/cvr/cvab169 33982064 PMC8477792

[24] Banerjee A Chen S Fatemifar G Zeina M Lumbers RT Mielke J. Machine learning for subtype definition and risk prediction in heart failure, acute coronary syndromes and atrial fibrillation: systematic review of validity and clinical utility. BMC Med. (2021) 19:85. 10.1186/s12916-021-01940-7 33820530 PMC8022365

[25] Ambale-Venkatesh B Yang X Wu CO Liu K Hundley WG McClelland R. Cardiovascular event prediction by machine learning: the multi-ethnic study of atherosclerosis. Circ Res. (2017) 121:1092–101. 10.1161/CIRCRESAHA.117.311312 28794054 PMC5640485

[26] Attia ZI Noseworthy PA Lopez-Jimenez F Asirvatham SJ Deshmukh AJ Gersh BJ. An artificial intelligence-enabled ECG algorithm for the identification of patients with atrial fibrillation during sinus rhythm: a retrospective analysis of outcome prediction. Lancet. (2019) 394:861–7. 10.1016/S0140-6736(19)31721-0 31378392

[27] Bundy JD Heckbert SR Chen LY Lloyd-Jones DM Greenland P. Evaluation of risk prediction models of atrial fibrillation (from the Multi-Ethnic Study of Atherosclerosis [MESA]). Am J Cardiol. (2020) 125:55–62. 10.1016/j.amjcard.2019.09.032 31706453 PMC6911821

[28] Christopoulos G Graff-Radford J Lopez CL Yao X Attia ZI Rabinstein AA. artificial intelligence-electrocardiography to predict incident atrial fibrillation: a population-based study. Circ Arrhythm Electrophysiol. (2020) 13:e009355. 10.1161/CIRCEP.120.009355 33185118 PMC8127001

[29] Hill NR Ayoubkhani D McEwan P Sugrue DM Farooqui U Lister S. Predicting atrial fibrillation in primary care using machine learning. PLoS ONE. (2019) 14:e0224582. 10.1371/journal.pone.0224582 31675367 PMC6824570

[30] Kim IS Yang PS Jang E Jung H You SC Yu HT. Long-term PM2. 5 exposure and the clinical application of machine learning for predicting incident atrial fibrillation. Sci Rep. (2020) 10:16324. 10.1038/s41598-020-73537-8 33004983 PMC7530980

[31] Hill NR Sandler B Mokgokong R Lister S Ward T Boyce R. Cost-effectiveness of targeted screening for the identification of patients with atrial fibrillation: evaluation of a machine learning risk prediction algorithm. J Med Econ. (2020) 23:386–93. 10.1080/13696998.2019.1706543 31855091

[32] Khurshid S Friedman S Reeder C Di Achille P Diamant N Singh P. Electrocardiogram-based deep learning and clinical risk factors to predict atrial fibrillation. Circulation. (2021). 10.1161/circ.144.suppl_1.12922 34743566 PMC8748400

[33] Raghunath S Pfeifer JM Ulloa-Cerna AE Nemani A Carbonati T Jing L. Deep neural networks can predict new-onset atrial fibrillation from the 12-lead ECG and help identify those at risk of atrial fibrillation-related stroke. Circulation. (2021) 143:1287–98. 10.1161/CIRCULATIONAHA.120.047829 33588584 PMC7996054

[34] Boon KH Khalil-Hani M Malarvili MB. Paroxysmal atrial fibrillation prediction based on HRV analysis and non-dominated sorting genetic algorithm III. Comput Methods Programs Biomed. (2018) 153:171–84. 10.1016/j.cmpb.2017.10.012 29157449

[35] Xin Y Zhao Y. Paroxysmal atrial fibrillation recognition based on multi-scale wavelet alpha-entropy. Biomed Eng Online. (2017) 16:121. 10.1186/s12938-017-0406-z 29061181 PMC5654099

[36] Boon KH Khalil-Hani M Malarvili MB Sia CW. Paroxysmal atrial fibrillation prediction method with shorter HRV sequences. Comput Methods Programs Biomed. (2016) 134:187–96. 10.1016/j.cmpb.2016.07.016 27480743

[37] Ebrahimzadeh E Kalantari M Joulani M Shahraki RS Fayaz F Ahmadi F. Prediction of paroxysmal atrial fibrillation: a machine learning based approach using combined feature vector and mixture of expert classification on HRV signal. Comput Methods Programs Biomed. (2018) 165:53–67. 10.1016/j.cmpb.2018.07.014 30337081

[38] Parsi A Glavin M Jones E Byrne D. Prediction of paroxysmal atrial fibrillation using new heart rate variability features. Comput Biol Med. (2021) 133:104367. 10.1016/j.compbiomed.2021.104367 33866252

[39] Castro H Garcia-Racines JD Bernal-Norena A. Methodology for the prediction of paroxysmal atrial fibrillation based on heart rate variability feature analysis. Heliyon. (2021) 7:e08244. 10.1016/j.heliyon.2021.e08244 34765772 PMC8569481

[40] Chesnokov YV. Complexity and spectral analysis of the heart rate variability dynamics for distant prediction of paroxysmal atrial fibrillation with artificial intelligence methods. Artif Intell Med. (2008) 43:151–65. 10.1016/j.artmed.2008.03.009 18455375

[41] Tiwari P Colborn KL Smith DE Xing F Ghosh D Rosenberg MA. Assessment of a machine learning model applied to harmonized electronic health record data for the prediction of incident atrial fibrillation. JAMA Netw Open. (2020) 3:e1919396. 10.1001/jamanetworkopen.2019.19396 31951272 PMC6991266

[42] Grout RW Hui SL Imler TD El-Azab S Baker J Sands GH. Development, validation, and proof-of-concept implementation of a two-year risk prediction model for undiagnosed atrial fibrillation using common electronic health data (UNAFIED). BMC Med Inform Decis Mak. (2021) 21:112. 10.1186/s12911-021-01482-1 33812369 PMC8019173

[43] Han L Askari M Altman RB Schmitt SK Fan J Bentley JP. Atrial fibrillation burden signature and near-term prediction of stroke: a machine learning analysis. Circ Cardiovasc Qual Outcomes. (2019) 12:e005595. 10.1161/CIRCOUTCOMES.118.005595 31610712 PMC8284982

[44] Lai D Bu Y Su Y Zhang X Ma CS. Non-standardized patch-based ECG lead together with deep learning based algorithm for automatic screening of atrial fibrillation. IEEE J Biomed Health Inform. (2020) 24:1569–78. 10.1109/JBHI.2020.2980454 32175879

[45] Breiman L. Random forests. Mach Learn. (2001) 45:5–32. 10.1023/A:1010933404324

[46] Breiman L. Statistical modeling: the two cultures (with comments and a rejoinder by the author). Stat Sci. (2001) 16:199–231. 10.1214/ss/1009213726

[47] Ishwaran H Kogalur UB Blackstone EH Lauer MS. Random survival forests. Ann Appl Stat. (2008) 2:841–60. 10.1214/08-AOAS169

[48] Inohara T Shrader P Pieper K Blanco RG Thomas L Singer DE. Association of of atrial fibrillation clinical phenotypes with treatment patterns and outcomes: a multicenter registry study. JAMA Cardiol. (2018) 3:54–63. 10.1001/jamacardio.2017.4665 29128866 PMC5833527

[49] Inohara T Piccini JP Mahaffey KW Kimura T Katsumata Y Tanimoto K. A cluster analysis of the Japanese Multicenter Outpatient Registry Of Patients With Atrial Fibrillation. Am J Cardiol. (2019) 124:871–8. 10.1016/j.amjcard.2019.05.071 31350002

[50] Pimor A Galli E Vitel E Corbineau H Leclercq C Bouzille G. Predictors of post-operative cardiovascular events, focused on atrial fibrillation, after valve surgery for primary mitral regurgitation. Eur Heart J Cardiovasc Imaging. (2019) 20:177–84. 10.1093/ehjci/jey049 29608669

[51] Carbone V Guarnaccia F Carbone G Battista Zito G Oliviero U Soreca S. Gender differences in the 12-lead electrocardiogram: clinical implications and prospects. Ital J Gender-Specific Med. (2020) 6:126–41. 10.1723/3432.34217

[52] Laureanti R Conte G Corino VDA Osswald S Conen D Roten L. Sex-related electrocardiographic differences in patients with different types of atrial fibrillation: Results from the SWISS-AF study. Int J Cardiol. (2020) 307:63–70. 10.1016/j.ijcard.2019.12.053 31959406

[53] Lau F Antonio M Davison K Queen R Devor A. A rapid review of gender, sex, and sexual orientation documentation in electronic health records. J Am Med Inform Assoc. (2020) 27:1774–83. 10.1093/jamia/ocaa158 32935124 PMC7671624

[54] Goldenberg I Erath JW Russo AM Burch AE Assmus B Bonderman D. Sex differences in arrhythmic burden with the wearable cardioverter-defibrillator. Heart Rhythm. (2021) 18:404–10. 10.1016/j.hrthm.2020.11.025 33248269

[55] Denton M Prus S Walters V. Gender differences in health: a Canadian study of the psychosocial, structural and behavioral determinants of health. Soc Sci Med. (2004) 58:2585–600. 10.1016/j.socscimed.2003.09.008 15081207

[56] Belle V Papantonis I. Principles and Practice of Explainable Machine Learning. Fron in Big Data. (2021) 4:688969. 34278297 10.3389/fdata.2021.688969 PMC8281957

[57] Stahl BC. Ethical Issues of AI. Artificial Intelligence for a Better Future. SpringerBriefs in Research and Innovation Governance. Springer, Cham. (2021). 35–53.

[58] Vidgen B Derczynski L. Directions in abusive language training data, a systematic review: Garbage in, garbage out. PLoS ONE. (2021) 15:e0243300. 10.1371/journal.pone.0243300 33370298 PMC7769249

